# Specialism in Dentistry

**Published:** 1895-04

**Authors:** 


### Editorial.

### SPECIALISM IN DENTISTRY.

It is probable that had the medical teacher of fifty years ago been told that in a half-century the practice of medicine would be divided into many subdivisions, each containing a body of men specially trained for a given work, he would have turned an incredulous ear and declared it impossible. Even as late as twenty-five years ago the specialist in medicine was regarded with as much disfavor as was the dentist, and looked upon as only one remove from the empirical pretender of the period. In the evolution of things these facts are forgotten, and only remembered by those whose experience sharpens the memory. The present generation. know next to nothing of this, hardly as history, yet it was a familiar fact at the period named. The man of special work has become the most active and the most valuable in medicine, while the general practitioner may have lost nothing of his prestige, but has gained in proportion to the progress made in special investigations.

This passing thought over general medicine leads to the question, Will dentistry that had an independent origin and development pass from a separate profession to that of a special branch? That this evolution is in progress, and rapidly so, no one can dispute who is familiar with the facts. It is not likely that the dentist of the future will be educated first in medicine and subsequently in dentistry. Certainly it is hoped this day will never come, for it would truly mean a change in the entire work of dentistry, with very questionable results as to practice. There should and doubtless will be a gradual development along medical lines of study, the curriculum being steadily advanced until it comprises all the essential branches of medical instruction. This is already an accomplished fact in the university departments throughout the country, and the course of study is steadily increasing to an extent not appreciated by those unfamiliar with the work. That this course must be followed by the independent schools is certain; for if not, then they must drop out of the contest. The lengthening of the course in these to correspond with the university sessions will probably determine this question in a very few years.

The next and correlated thought suggested is, What will be the result of this inevitable change?

Dentistry in its origin necessarily embraced almost entirely the mechanical idea. The purely professional had no place, and could not have had under then existing conditions. Beyond the occasional filling and the manufacture of artificial teeth, the dentist of the thirties made no attempt to advance. The professional side has developed slowly but surely, until now, near the close of the nineteenth century, we are brought face to face with the fact that to continue as it has is an impossibility, especially in the large centres of activity.

If, then, this be true, and it is difficult to regard it in any other light, it becomes the part of wisdom to direct thought in this direction, that in the final subdivisions there may be, at least, an attempt to improve these special branches that they may, in no degree, be inferior, but that each in its way may be worthy of entire respect.

It is clear that the twentieth century dentist will be occupied, not, as now, with all sorts of work, but will be with one of the following branches: pathology and therapeutics connected with general operations in the mouth; the surgical treatment of cases in the oral cavity; the preparation and insertion of artificial dentures; the preparation of metal crowns and improved bridge-work, and the extraction of teeth.

That this is not a utopian idea is apparent from the fact that this is already accomplished, in a large degree, in our cities. The dentist there, as a rule, has long since ceased to make the teeth he inserts. The surgery has passed into the hands of special workers. The crown and bridge-work, especially the latter, is in the hands of the specialist to a large extent. The processes of filling, treatment, etc., belong to another class; and extraction has almost entirely passed out of the hands of the general practitioner.

This is the present status, but is it satisfactory? In many respects the answer can be in the affirmative, in others not.

The great defect, it seems to us, exists in the mechanical branch. We have many very capable specialists in the mere mechanical details. They have attained the perfection of the jeweller's art, but they can go no further. Their work ceases here to be taken up by the general operator, neither working in entire harmony with the other, and both operating without much regard to conditions necessary to make an artistic piece of work. This specialty should be made an art worthy the life-work of any individual. The time is past for the horrible distortions met with. The prosthetic specialist, instead of being merely the skilful mechanic, should make the facial characteristics a study, have some knowledge of temperaments, study the rules of articulation, in a word, combine in his work all that is meant by the word artistic. He should be given the entire management of the insertion of an artificial denture, and thus bring it to a perfection that "art will conceal art," and not, as now, make it the most prominent feature of the work. In this way only, it seems to us, can this important specialty receive the attention it must ever demand. It should be evident that a dentist in full practice has not the time to look after artificial work of any kind; and if he has not the time, it cannot be done with that perfection and attention to artistic details most to be desired. The man who undertakes to do everything will, like the "jack of all trades," end in doing nothing well.

The views on mechanical dentistry apply with equal force to other branches of our work.

The extension of treatment in dental pathology has become so important and, in degree, intricate, that the ordinary dentist is entirely inadequate to meet it. The same applies to the work of the surgeon, a growing and most important work in dentistry.

The foregoing thoughts are given more as suggestions, and without any expectation of a speedy fulfilment; but it seems to us the dentist of the coming time should set his house in order and prepare for the work best adapted to his tastes and mental qualifications.

